# 

**Published:** 1847-09

**Authors:** 


### CONTENTS

OF NO. III.

### ORIGINAL COMMUNICATIONS.

### ESSAYS AND CASES.

ART. I.—Notes on Medical Matters and Medical Men in Paris. By DAVID W. YANDELL, M.D., of Louisville, Ky. .........185

ART. II.—The Black Death in Florence, translated from the Italian of Boccacio. By WM. M. BOLING, M.D., of Montgomery, Alabama ........................................209

ART. III.—A Case of Vesico-Vaginal Fistula remedied by Caustic. By ELAM W. HARRIS, M.D., of Elm Wood, Cape Girardeau county, Missouri.................................217

ART. IV.—A Case of Delirium Tremens induced by the inordinate Use of Tobacco. By WM. A. GORDON, M.D., of Harrisburg, Missouri.......................................219

### REVIEWS.

ART. V.—Observations on Aneurism, and its Treatment by Compression. By O'BRIEN BELLINGHAM, M. D., Edin.; Fellow of and Professor in the School of the Royal College of Surgeons in Ireland; Licentiate of the Royal College of Edinburgh, and one of the Surgeons to St. Vincent's Hospital. London: John Churchill. 1847.................221

### SELECTIONS FROM AMERICAN AND FOREIGN JOURNALS.

Observations on Dysmenorrhœa.......................................245
On the Varieties of Headache........................................250
On Latent Pneumonia.................................................252
Variola, Vaccinia, Varioloid, and Varicella.........................253

Treatment of Epistaxis by Insufflations of Alum.................254
Comparative Utility of the Bromide and Iodide of Potassium, in the Treatment of Secondary and Tertiary forms of Syphilis...254
Effects of Sudden Changes of Temperature.....................257
Pathology and Treatment of Croup.............................258
Ship Fever in Canada.........................................260
Munificence of the King of Prussia for Medical Services..........262
Method of Administering Quinine..............................262

ORIGINAL INTELLIGENCE.

Traveling Letters from the Senior Editor..............263, 270
Medical Reform.............................................274
On the Varieties of Headache................................275
Ranking's Half-Yearly Abstract...............................276
Law Department of the University of Louisville.................276
Medical Changes and Appointments............................276

